# Factors Influencing Virtual Reality Sickness in Emergency Simulation Training

**DOI:** 10.1007/s40670-024-02102-z

**Published:** 2024-07-05

**Authors:** Riyadh Firdaus, Aida Rosita Tantri, Sidharta Kusuma Manggala

**Affiliations:** Department of Anesthesiology and Intensive Care, Faculty of Medicine, Universitas Indonesia – Cipto Mangunkusumo Hospital, Jakarta, Indonesia

**Keywords:** Virtual Reality, Emergency Simulation, Cybersickness, Learning Experiences

## Abstract

**Introduction:**

Virtual reality-based simulation is an educational tool that has been proven to increase participants’ self-perceived, confidence, and skill. However, the use of VR is associated with virtual reality sickness (VRS). The purpose of this study is to determine related factors of VRS in an emergency setting simulation-based training, hence providing information and mitigation plan to enhance and optimize learning outcomes.

**Method:**

We developed multiplayer VR simulation in Traumatic Brain Injury and Local Anaesthetic Systemic Toxicity emergency. Seventy-five medical doctors voluntarily partake in the VR simulation. Throughout the simulation and its aftermath, participants were carefully monitored and observed. Additionally, they were questioned regarding their experience of VRS using the Simulation Sickness Questionnaire (SSQ) instrument.

**Result:**

The incidence of virtual reality sickness was found to be 57.3% and is significantly associated with male gender, myopia, astigmatism, and the use of stationary VR mode (*p*<0.05). The mean SSQ score for nausea, oculomotor, disorientation, and total score component is 5.97 (standard deviation (SD): 6.4), 6.26 (SD 6.5), 125.6 (SD 132), and 9.03 (SD 9.5), respectively. There were 16% of participant experiencing severe symptoms of VRS.

**Conclusion:**

Male gender, myopia, astigmatism, and the use of stationary VR mode were related with incidence of virtual reality sickness. VR activities in either room-scale or walking mode and appropriate correction of any refractive disorders are pivotal to prevent VRS in VR simulation training.

## Introduction

As the artificial intelligence industry continues to gain momentum, there is a rising trend of integrating virtual reality (VR) technology into diverse sectors, including medical education in the form of high fidelity training simulation [1]. Studies show that virtual simulation-based training is effective in teaching procedural medical skills [2–9]. However, during VR experiences, some users may suffer from troublesome symptoms that are similar to motion sickness, termed virtual reality sickness. Motion sickness can potentially affect learning ability. When an individual experiences virtual reality sickness, they may feel nauseous, dizzy, and uncomfortable, which can lead to a range of cognitive and physical effects that may impact the ability to learn [10].

People rely on different sensory organs to understand orientation and movement. The vestibular, visual, and proprioceptive senses are particularly important in forming a unified perception of self-motion in three-dimensional surroundings. Harmonious processing of all these sensory inputs results in effortless and accurate recognition of position and movement in space. Motion sickness can arise when there is a disparity between visual information and dynamic vestibular input, leading to sensory conflicts between the incoming signals about the current state. When the primary sensory input triggering motion sickness is the visual stimuli, the term visually induced motion sickness (VIMS) is used. The VIMS, depending on the situation, may be called gaming sickness, simulator sickness, cinerama sickness, or virtual reality sickness (VRS) [1, 11, 12]. The VRS is described in the literature by a variety of terms, including cybersickness, simulator sickness, VIMS, and VR-induced symptoms. Prevalence of VRS during or post VR experience has been reported to be as high as 80% [13]. Various factors, including attributes of the intervention itself (such as content and hardware utilized) and individual subjective characteristics, contribute to the determination of VRS symptom severity. According to prior studies, the factors that affect VRS can be classified into VR hardware (the weight, the comfortability, the user interface), the content of the VR simulation (technicality, exposure duration, visual simulation, locomotion), and human-related aspects (prior experiences, eye-related condition, screen time, age, gender) [1, 13].

One of the significant factors influencing VRS is the user’s familiarity with the head-mounted display (HMD) within the virtual environment and the quality of the simulation training content. Furthermore, in the context of emergency simulation training, various locomotion challenges are encountered. The implementation of appropriate locomotion techniques in VR is imperative to ensure a high level of immersion and a sense of presence while navigating and engaging with the virtual environment [14]. In emergency situations, healthcare professionals are required to possess knowledge and skill concerning machine assembly, equipment operations, vital signs monitoring, communication, and a range of medical competencies. In emergency setting, time is critical and delays in responding or making decisions can have significant consequences in emergency situations. These issues can add more burden to the locomotion challenge they already possessed with virtual reality training. Consequently, visual acuity, spatial awareness, effective communication, and seamless coordination play essential roles and are indispensable for a comprehensive and high-quality training in virtual reality simulation experience for emergency [15, 16].

This study is a part of VR-TBI (Virtual Reality-Traumatic Brain Injury) and VR-LAST (Virtual Reality-Local Anaesthetic Systemic Toxicity) project. The project is developed by the Department of Anaesthesiology and Intensive Care, Faculty of Medicine, Universitas Indonesia to evaluate the use of VR technology in emergency simulation training. The purpose of this study is to determine the incidence of VRS in an emergency setting simulation-based training and the related factors, hence providing information and mitigation plan of the virtual reality-related side effects to enhance and optimize learning outcomes.

## Method

### Study Design

This study is a prospective observational study with cross-sectional design that took place in April–June 2023. The target population of our study comprises individuals who voluntarily participate in virtual reality-based simulation training. The study populations, during the designated period, were confined to doctors and doctors in training affiliated with the Faculty of Medicine at the Universitas Indonesia.

### Simulation Sickness Questionnaire

The Simulation Sickness Questionnaire (SSQ) is used to measure the VRS as the study outcome. It was first derived from the 1983 Motion Sickness Questionnaire (MSQ) that was originally used to evaluate motion sickness in various forms of transportation. In 1993, Kennedy et al. reported the SSQ for the first time, proposing 16 items derived from MSQ that was divided into three categories: nausea, oculomotor, and disorientation (Table 1) [17].

**Table 1:** Symptoms in SSQ

**SSQ items**	**Nausea**	**Oculomotor**	**Disorientation**
General discomfort	1	1	
Fatigue		1	
Headache		1	
Eyestrain		1	
Difficulty focusing		1	1
Increased salivation	1		
Sweating	1		
Nausea	1		1
Difficulty concentrating	1	1	
Fullness of head			1
Blurred vision		1	1
Dizzy (eyes open)			1
Dizzy (eyes closed)			1
Vertigo			1
Stomach awareness	1		
Burping	1		
Total	(1)	(2)	(3)

*SSQ* Simulation Sickness Questionnaire

Formulas for scoring each component of the SSQ were proposed as Table 2. It is possible to obtain four SSQ scores: nausea, oculomotor, disorientation, and total. The score of each category is defined as the sum of its symptom scores multiplied by a constant scaling factor. Based on a large sample of SSQ data gathered from military pilots, it is suggested that total scores can be associated with negligible (<5), minimal [5–10], significant [10–15], and concerning [15–20], and bad (>20) symptoms [18].

**Table 2:** Computation of SSQ scores

**SSQ components**	**Computation**
Nausea	(1) × 9.54
Oculomotor	(2) ×7.58
Disorientation	(3) × 13.92
Total	[(1) + (2) + (3)] × 3.74

*SSQ* Simulation Sickness Questionnaire

#### The Procedure

Participants willingly volunteered to partake in the VR simulation scenario. Prior to the trial, they were provided with a comprehensive explanation of the sequence of the VR simulation and instructed on how to proceed during the experience. Throughout the simulation and its aftermath, participants were carefully monitored and observed for any changes in behaviour. Additionally, they were questioned regarding their experience of VRS using the SSQ instrument.

#### The Virtual Reality Simulation-Based Training

**Fig. 1:**
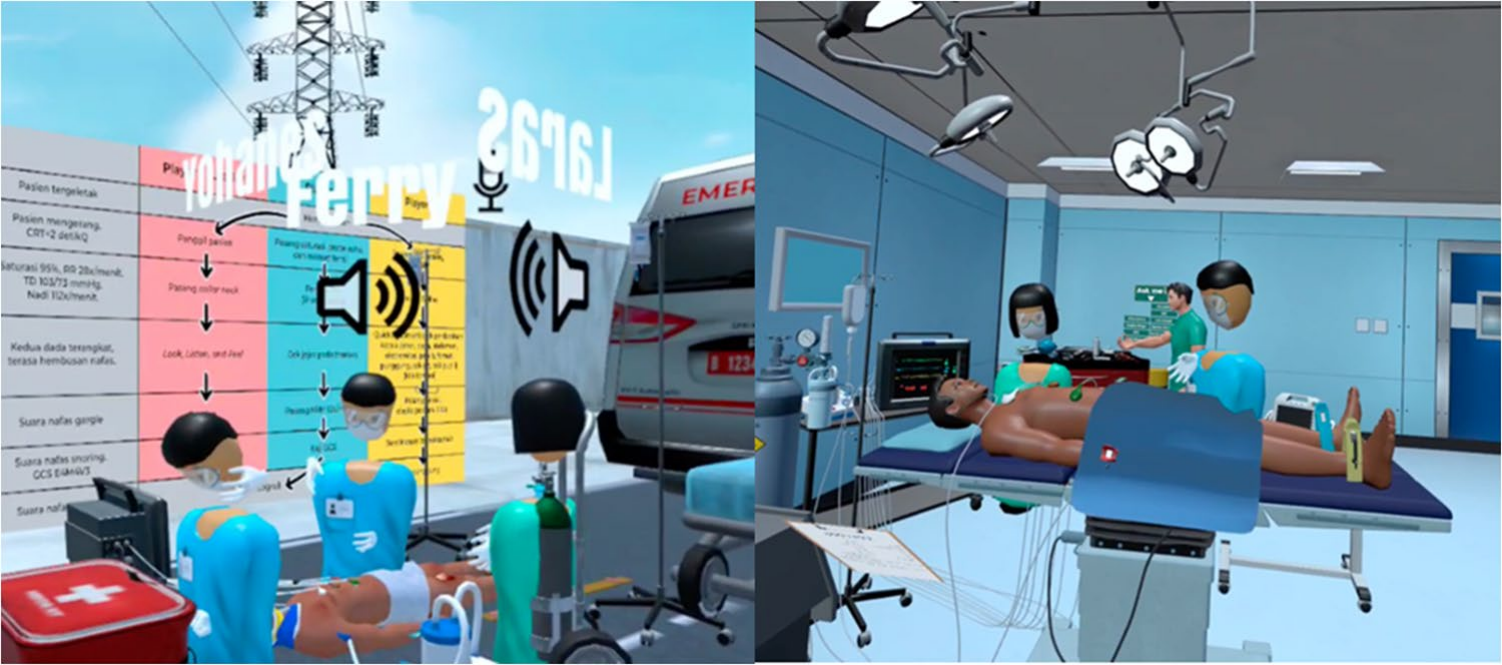
The scenario inside VR-TBI (left) and VR-LAST (right)

There were two training scenarios provided within this study: the traumatic brain injury and the local anaesthetic systemic toxicity (Fig. 1). Each participant can choose one scenario to enrol based on their preference. The traumatic brain injury (TBI) scenario consists of three settings; prehospital setting, ambulance setting, and emergency room setting. The player took role as doctor who treat the emergency patient. If the sequence is properly followed and player does not encounter problems, the TBI scenario can be completed in 20–30 min. The local anaesthetic systemic toxicity (LAST) scenario consists of 15–20 min single setting scene in the operating room. The players take role as resident doctors treating patient who experienced seizure after epidural administration. Each simulation is structured with checklists of the tasks players need to accomplish by the end of the simulation.

#### The Virtual Reality Hardware

**Fig. 2:**
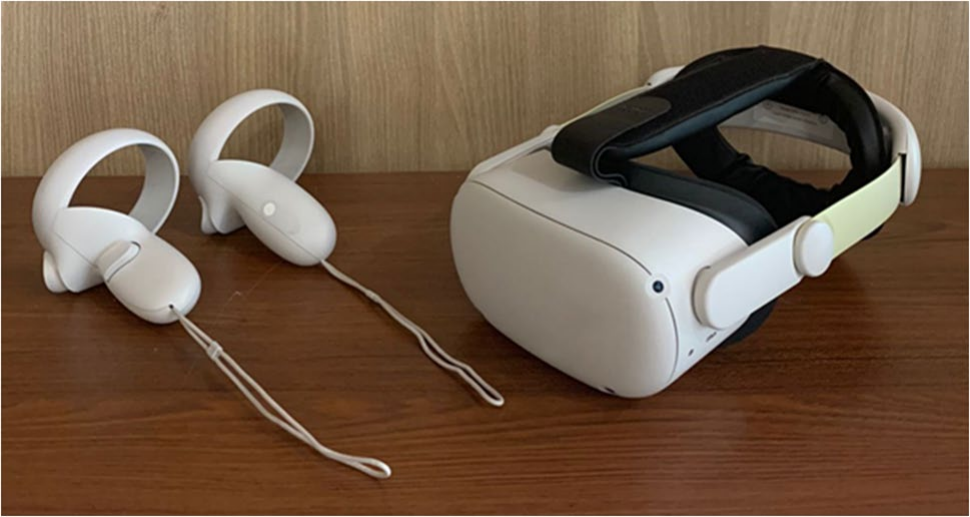
Virtual reality headset

**Fig. 3:**
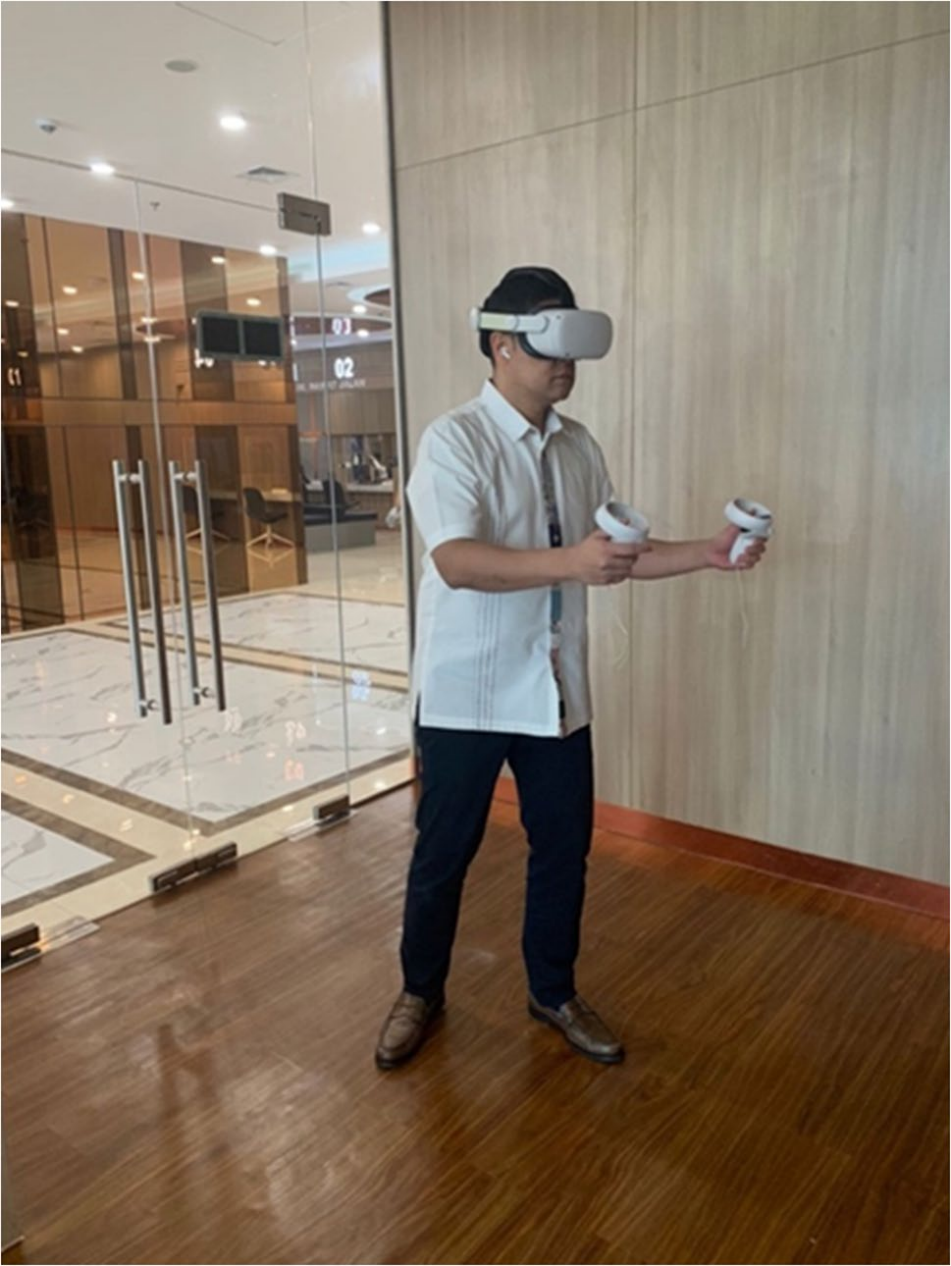
A doctor playing inside the VR with VR headset in stationary mode

In both scenarios, we employed the Oculus Quest 2 VR headset, which weighs approximately 503 g (17.7 ounces). The Occulus Quest 2 is consists of one VR headset with head-mounted display and two controllers for right and left hands (Fig. 2). The scenarios were executed with two distinct mode options: “stationary” and “room scale”. In the room scale mode, players had the opportunity to physically walk within a designated physical area, allowing them to make real-world motions while fully immersing themselves in the virtual environment. Conversely, in the stationary mode, users were confined to a fixed 3 × 3 feet square area (Fig. 3).

## Result

The study involved 75 students who participated voluntarily. Table 3 shows the baseline characteristics of participants. The majority of the participants had no prior experience with VR. During the study, several factors were observed that could influence the VRS among the participants. These factors included a history of vertigo (1.3%), myopia (34.7%), astigmatism (14.7%), wearing glasses (29.3%), and screen time. The median screen time among participants is 10 h. Additionally, another factor associated with VRS was the type of VR mode used. Among the voluntary participants, roughly equal proportions of them used room scale (52%) and stationary (48%) VR mode. There were 57.3% participants participating in TBI scenario, and the rest 42.7% participated in LAST scenario.

**Table 3:** Baseline characteristics

**Characteristics**	**Frequency (percentage)/median (Min-Max)**
**Gender**	
Female	37 (49.3)
Male	38 (50.7)
**Occupation**	
Resident	32 (42.7)
General practitioner	12 (16)
Medical student	31 (41.3)
**VR experience**	
Never	61 (81.3)
Once	9 (12)
More than once	5 (6.7)
Age^*^	27 (20–32)

*Data is presents as median (min-max)

**Fig. 4:**
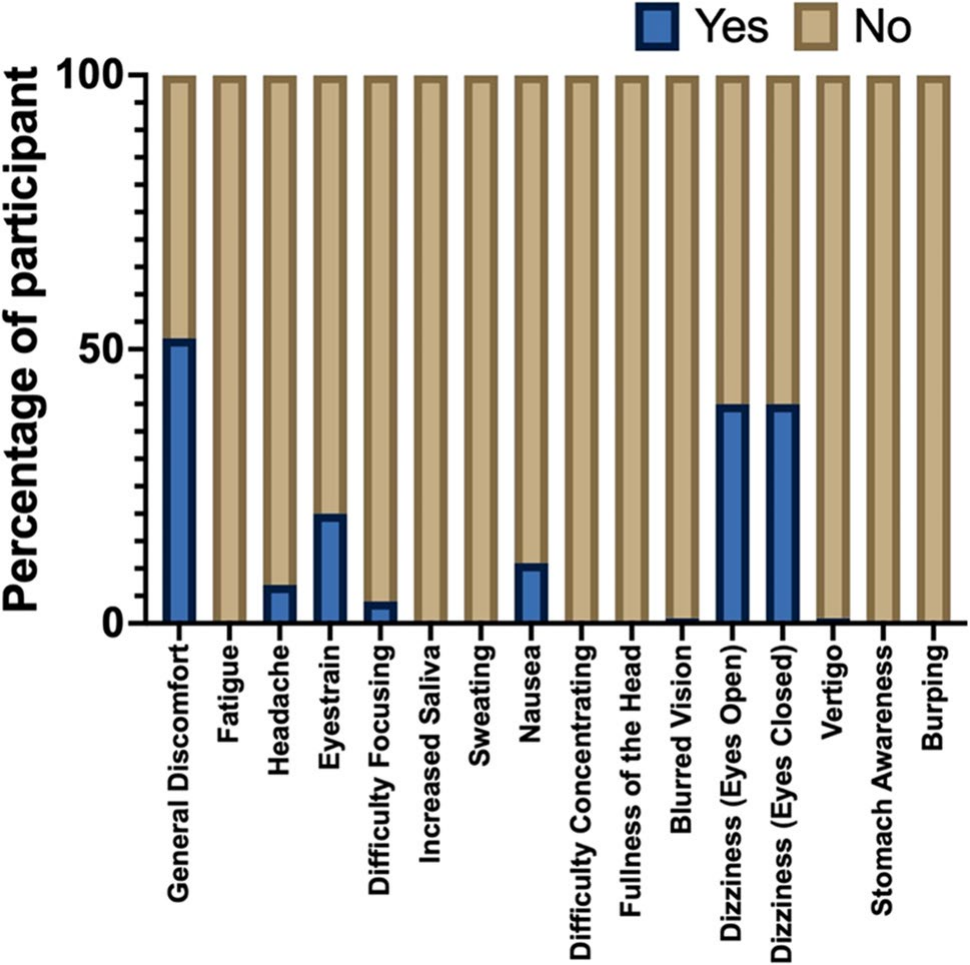
Symptoms from SSQ

The presence of VRS is acknowledged if the sample encounters any of the symptoms mentioned in the SSQ. There were 43 (57.3%) participants experiencing VRS in the study with total SSQ mean of 9 (SD 9.5) that is considered minimal symptoms. Figure 4 shows the frequency of symptoms from SSQ that the participants experienced.

**Fig. 5:**
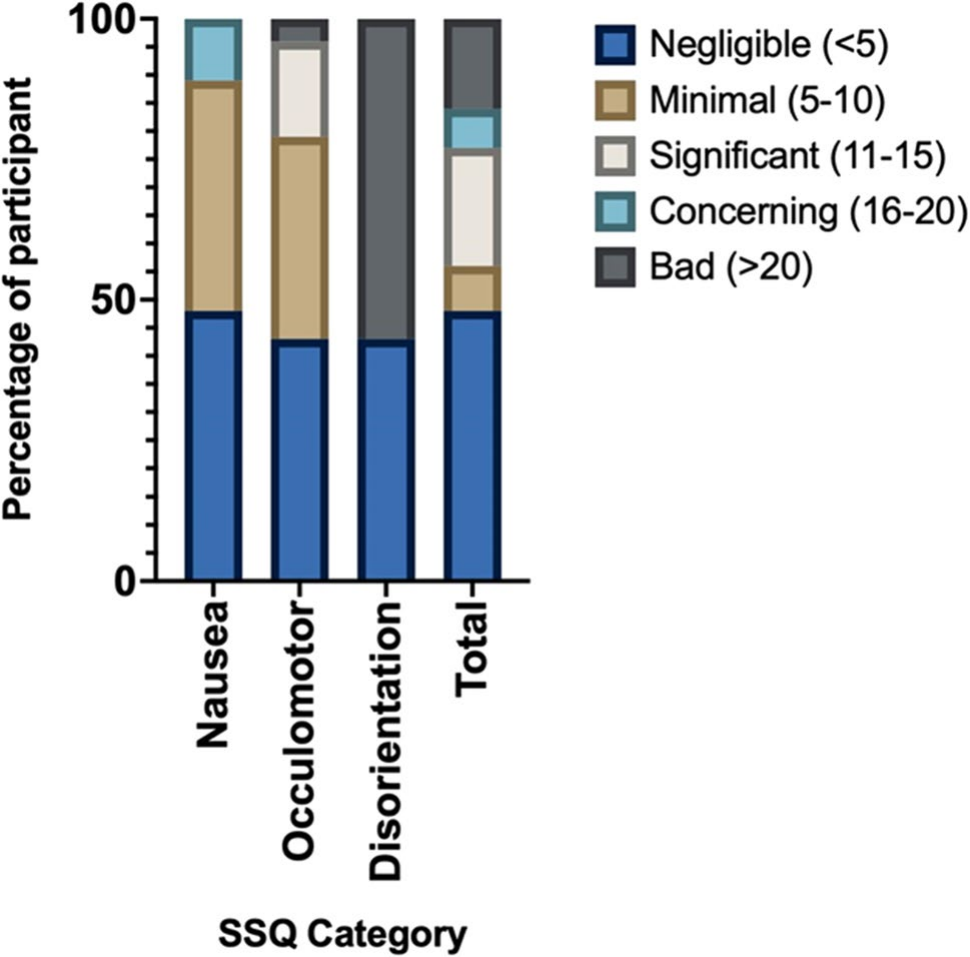
Sickness severity on each SSQ category

Out of 75 participants, the mean score for nausea, oculomotor, disorientation, and total score is 5.97 (SD 6.4), 6.26 (SD 6.5), 125.6 (SD 132), and 9.03 (SD 9.5) respectively. The SSQ score can be classified into 5 categories based on the severity, and it is shown in Fig. 5.

Of the observed factors, we tried to measure the contribution of the factors to the VRS with logistic regression. The OR has been calculated as adjusted OR while controlling for one or more additional variables that could confound the results. This adjustment is done to isolate the effect of the primary variable of interest. Adjusted OR of these factors, gender, astigmatism, and VR mode are associated with VRS, as can be seen in Table 4.

**Table 4:** Virtual reality sickness factors

**Factor**	**VRS**		*p* value	Adjusted OR^*^ **(CI95%)**
	Negative	Positive		
**Gender**				
Female	22	15	**0.028**	2.82 (1.1–7.2)
Male	13	25		
**Vertigo history**				
No	32	42	1	-^#^
Yes	0	2		
**Myopia**				
No	25	24	**0.045**	2.8 (1.1–7.9)
Yes	7	19		
**Astigmatism**				
No	32	32	**0.002**	-^#^
Yes	0	11		
**Wearing glasses**				
No	26	27	0.082	2.56 (0.87–7.57)
Yes	6	16		
**VR mode**				
Room scale	24	15	**0.001**	5.6 (2.02–15.47)^*^
Stationary	8	28		
**Previous VR experience**				
Never	28	33	0.782	0.848 (0.26–2.71)
At least once	7	7		
**Screen time (hour)**	10.38	10.16	0.8	0.21 (−1.06–1.48)^+^

*Adjusted OR from logistic regression

^#^OR cannot be calculated due to one cell with 0 number

^+^Mean difference (standard deviation)

## Discussion

In this study, we observed that virtual reality sickness (VRS) affected 57.3% of the 75 participants (Fig. 4). This number aligns with findings from previous studies, which reported an incidence rate between 60 and 70% [13, 18–21]. The mean SSQ score in our study was 9 with a range of 0 to 29, indicating lower score than previous study by Saredakis et al. which reported a pooled mean of 28 with a range of 14.30 to 35.27 [19]. Among the SSQ components, disorientation scored the highest at 125.6, followed by oculomotor at 6.36, and nausea at 5.97 (Fig. 5). These results are consistent with previous research reporting pooled mean SSQ scores of 16.72, 17.09, and 23.5 for nausea, oculomotor, and disorientation, respectively [19]. According to Kenendy et al., total SSQ score of 9 indicates minimal symptoms [17, 18]. Although majority of the participants in our study experienced negligible to significant symptoms, there were 16% of them that experienced severe symptoms, and further mitigation plan should be prompted.

In the context of emergency setting simulations, healthcare professionals are mandated to possess not just the knowledge base, but a comprehensive technical skill set. This proficiency encompasses the continuous monitoring of hemodynamic changes subsequent to specific medical procedures like looking at the monitor over and over after performing a medical procedure. This well involved repetitive rotational movement of the neck. Other technical skill healthcare professionals need to understand are the assembly and operation of medical equipment, effective communication, and a diverse array of medical competencies, such as airway management, resuscitation, and the precise administration of pharmaceuticals. In an emergency scenario, temporal considerations are of paramount importance, as any delays in response time or decision-making may yield profound and potentially life-altering consequences. Moreover, the demanding nature of emergency setting simulations, characterized by intricate and occasionally repetitive actions, can engender VRS.

The disorientation component of the SSQ, encompassing symptoms such as difficulty focusing, nausea, fullness of the head, blurred vision, dizziness with eyes open or closed, and vertigo, exhibited a high occurrence of bad scores (>20) in 43 participants (57.3%) as seen in Fig. 5. Our research findings have illuminated that VRS, particularly of a severe degree, predominantly falls within the disorientation category. This observation implies that the performance of users engaged in simulation exercises may be disrupted and hindered, preventing them from effectively showcasing their genuine competencies during training simulations. This can further impact learning process. Kelly et al. discovered that virtual reality users are particularly susceptible to disorientation, especially when using locomotion interfaces lacking self-motion cues. To address this issue, incorporating environmental cues, such as boundaries, into the design of locomotion interfaces is crucial to reduce disorientation-related effects [22].

Our study found that hardware-related issues, specifically the VR mode, were associated with VRS. Participants using the stationary mode were five times more likely to experience VRS. This is in line with previous research indicating that nonstationary VR modes, especially physically walking mode, can help reduce the incidence of VR sickness [23]. Chang et al. classified VRS factors into three domains: hardware, content, and human factors. Hardware factors encompassed various VR device manipulations, including display type, display mode, time delay, and device weight. Content factors involved altering VR scenes or scenarios, such as graphics, task-related features, duration, and controllability. Human factors considered individual differences, such as age, gender, BMI, postural sway, previous VR experience, and eye-related measures like interpupillary distance, refractive error, and eye-hand coordination [1]. In Saredakis et al.’s study, the mean SSQ scores for nausea, oculomotor, and disorientation were 22.6, 22.4, and 28.5, respectively, in the stationary group, whereas in the walking group, they were 13.2, 15.3, and 18.5, respectively [19, 24].

Our findings revealed that male are more prone to VRS. This is the opposite of previous studies. Schmitz et al. found gender as an essential variable associated with motion sickness in VR systems [25]. Males were 2.8 times more likely to experience VRS than females. However, a large-scale meta-analysis reported no significant gender difference in VRS [19]. Former VR experience was believed to increase the likelihood of VRS [26]. Surprisingly, our study did not find any significant effect of previous VR experience on VRS. Further research is needed to fully comprehend the underlying pathophysiology of this phenomenon [27]. Young et al. discovered that ocular refraction disorders influenced motion sickness during head-mounted display experiences. Myopic and astigmatic participants showed significantly higher SSQ scores for nausea and disorientation. Our findings are aligned with these results, as we observed a higher incidence of VRS in participants with myopia and astigmatism.

This study has several limitations. Firstly, its cross-sectional design makes it difficult to establish causal relationships between factors. It would be better if further research could address the dose-response relationship between the amount of time spent in VR-based learning and development of fatigue. Conducting further research using cohort or randomized trials would be more suitable for addressing this issue. Secondly, the consecutive sampling method used may introduce bias as it is a nonprobability sampling technique. Thirdly, the lack of pre-treatment SSQ scores before participants engaged in the VR experience could lead to additional biases in interpreting the results.

We interviewed participants with simple preference questions, asking their opinion whether VR is potential for learning method in the near future and whether the VRS that they experienced hinder them for trying VR for educational purposes in the near future. All of the 75 people answered that VR has the potential for learning method, and only one of 75 people has chosen to avoid using VR in the near future due to the side effect that he experienced.

Based on the current report, we recommend using the room-scale mode as the locomotion interface for VR to minimize VRS. Additionally, participants with refractive disorders should receive proper treatment by wearing glasses for refractive correction before engaging in VR activities. These strategies are essential to ensure all participants have the best possible environment to enhance learning outcomes in VR-based simulation training for traumatic brain injury management.

## Conclusion

The incidence of virtual reality sickness was associated with male gender, myopia, astigmatism, and the use of stationary VR mode. To prevent virtual reality sickness during VR simulation training, it is essential to engage in VR activities in room-scale or walking mode and ensure appropriate correction of any refractive disorders. Future research should address the dose-response relationship between the amount of time spent in VR-based learning and development of virtual reality sickness.
